# Supplementary material for: Soil Type Influences Novel “Milpa” Isolates of Trichoderma virens and Aspergillus tubingensis That Promote Solubilization, Mineralization, and Phytoabsorption of Phosphorus in Capsicum annuum L
Source: J Fungi (Basel). 2022 Nov 8;8(11):1178. doi: 10.3390/jof8111178 (PMC9699344; doi:10.3390/jof8111178)
Supplement: Supplementary file 1 [file jof-08-01178-s001.zip › Supplementary material Zuniga-Silgado2022.pdf]

Supplementary material

Table S1. Georeferencing and physico-chemical characteristics of the studied soils

Georeferencing and physico-chemical characteristics of the studied soils		
**Soil**	Andisol	Vertisol
**Country**	Mexico	Mexico
**State**	Morelos	Morelos
**Municipality**	Huitzilac	Temixco
**Locality**	Tres Marías	Cuentepec
**Coordinates**	19º02'18''N 99º15'11''W; 2803 masl	18º51'36''N 99º19'29''W; 1480 masl
**Taxonomic order**	Andisol (Melanudand)	Vertisol (Aquerts)
**Sand (%)**	92	56
**Silt (%)**	4	24
**Clay (%)**	4	20
**Texture (Bouyoucos)**	A	FarA
**pH (w, 1:2.5)**	5.7	4.9
**M.O. (Walkley-Black) (%)**	11	3.0
**Ca (Ammonium acetate 1M) ($cmol_c$ kg^{-1})**	1.7	3.0
**Mg (Ammonium acetate 1M) ($cmol_c$ kg^{-1})**	0.1	1.7
**K (Ammonium acetate1M) ($cmol_c$ kg^{-1})**	0.05	0.16
**Na (Ammonium acetate1M) ($cmol_c$ kg^{-1})**	0.0	0.0
**Al (KCl 1M) ($cmol_c$ kg^{-1})**	0.0	1.6
**CIC effective ($cmol_c$ kg^{-1})**	1.85	6.5
**P Bray II (mg kg^{-1})**	1	34
**S (Calcium phosphate 0.008 M) (mg kg^{-1})**	6	2
**Fe (Olsen-EDTA) (mg kg^{-1})**	64	42
**Mn (Olsen-EDTA) (mg kg^{-1})**	1	53
**Cu (Olsen-EDTA) (mg kg^{-1})**	1	2
**Zn (Olsen-EDTA) (mg kg^{-1})**	1	3
**B Hot water) (mg kg^{-1})**	0.1	0.14
**NO_3 (Aluminum sulfate 0.025 M) (mg kg^{-1})**	3	1
**NH_4 (KCl 1M) (mg kg^{-1})**	18	38

Table S2. Detection of the type and concentration of OAs in different soils with strains (*T. virens* and *A. tubingensis*). The values are the mean of the concentration of acids (µg mL^{-1}) ± SE extrapolated from 250 mL samples of four different soil samples in their interaction with microorganisms A, B, C and D (n = 4). Bolded values depict the condition in which the highest value for that particular acid was found.

Soil	Microorganism	Pyruvic µg mL^{-1}	Fumaric µg mL^{-1}	Tartaric µg mL^{-1}	Succinic µg mL^{-1}	Malic µg mL^{-1}	Oxalic µg mL^{-1}	Citric µg mL^{-1}	Concentration P µg mL^{-1}
**Andisol**	***T. virens***	**0.422** ± 0.142	**0.002** ± 0.001	**0.496** ± 0.049	**0.002** ± 0.001	**0.058** ± 0.043	**0.006** ± 0.001	**0.003** ± 0.002	**29.187** ± 0.400
	A. tubingensis	**0.122** ± 0.070	**0.014** ± 0.001	**0.404** ± 0.238	**0.124** ± 0.041	**0.007** ± 0.014	**0.005** ± 0.000	0.000	**35.217** ± 0.0800
**Vertisol**	***T. virens***	**0.063** ± 0.008	**0.010** ± 0.000	**0.032** ± 0.019	**0.028** ± 0.003	**0.014** ± 0.003	**0.023** ± 0.001	**0.005** ± 0.000	**34.240** ± 0.209
	A. tubingensis	**0.049** ± 0.028	**0.052** ± 0.000	**1.834** ± 0.004	0.000	**0.024** ± 0.006	**0.062** ± 0.009	**0.005** ± 0.001	**36.369** ± 0.711
**Without Soil**	***T. virens***	0.000	**0.001** ± 0.000	0.000	0.000	**0.004** ± 0.003	**0.050** ± 0.032	0.000	**40.615** ± 0.883
	A. tubingensis	0.000	**0.002** ± 0.001	0.000	**0.036** ± 0.012	**0.032** ± 0.046	**0.005** ± 0.001	0.000	**37.505** ± 0.350

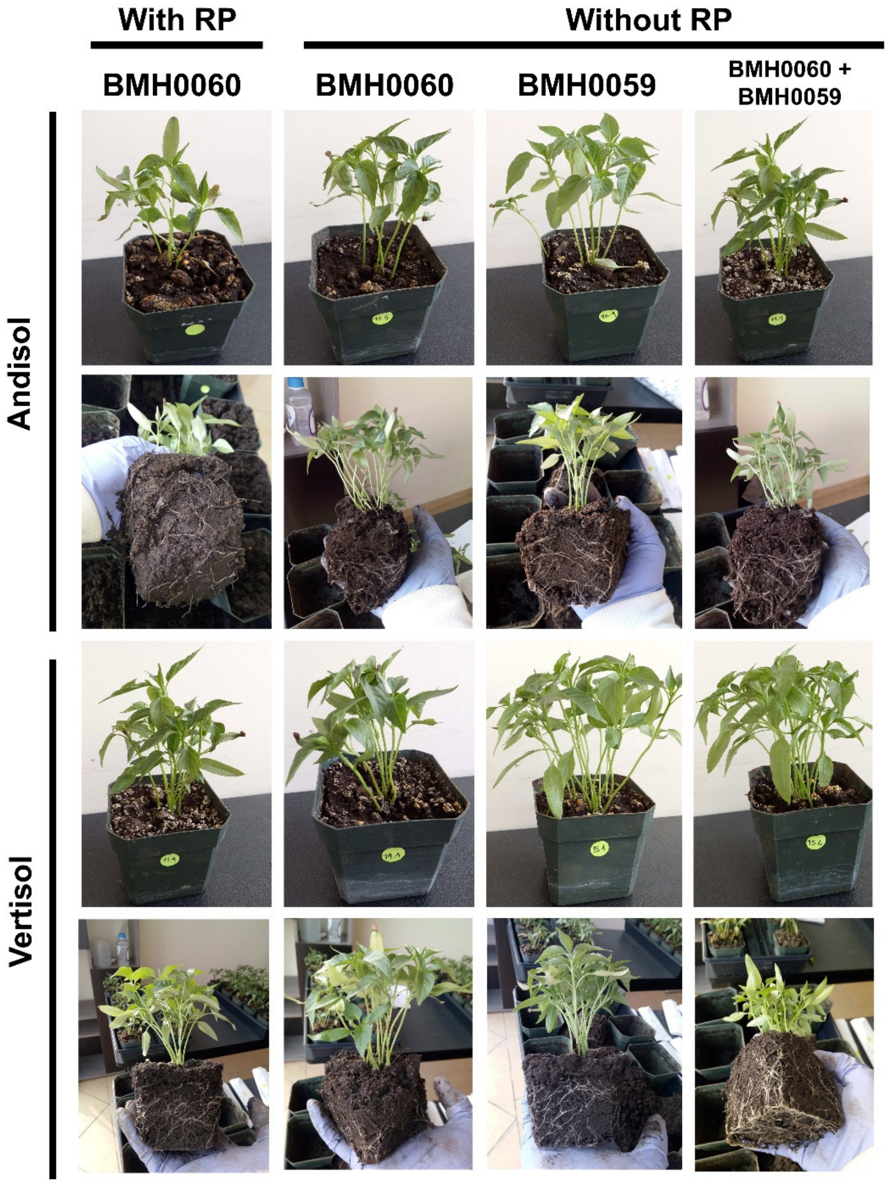

Figure S1. Representative images of the biometric parameters in *C. annuum* L inoculated with and without RP (RP^+ and RP^-), adding simple and dual treatments with *T. virens* (PMF) and/or *A. tubingensis* (PSF) 7×10^6 spores mL^{-1}), grown in two soils Andisol (And) and Vertisol (Ver).

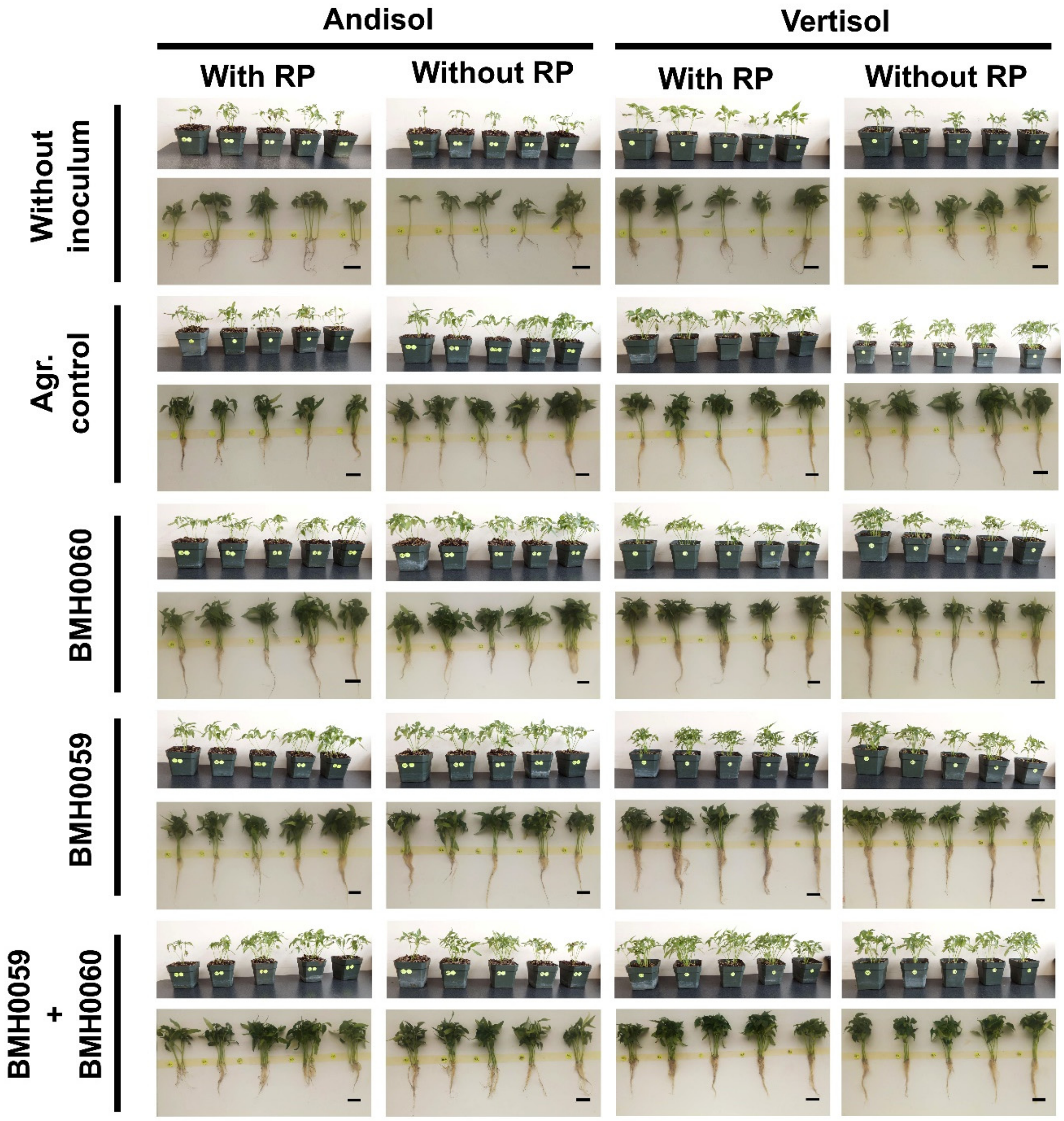

Figure S2. Representative images of biometric parameters in *C. annuum* L inoculated with and without RP (RP^+ and RP^-), adding simple and dual treatments with *T. virens* (PMF) and/or *A. tubingensis* (PSF) $7x10^6$ spores mL^{-1}), grown in two soils Andisol (And) and Vertisol (Ver)
